# Supplementary material for: Oxidative Stress Response of Probiotic Strain Bifidobacterium longum subsp. longum GT15
Source: Foods. 2023 Sep 7;12(18):3356. doi: 10.3390/foods12183356 (PMC10530004; doi:10.3390/foods12183356)
Supplement: Supplementary file 1 [file foods-12-03356-s001.zip › foods-2506949-supplementary.pdf]

Table S1. List of genes and their products with antioxidant functionality of bifidobacteria

The product encoded by the gene	Antioxidant functionality	Gene	Strain	Reference
Ferroxidase	Iron chelation activity	BL01563	*B. longum* NCC2705	[Kwak et al.,2016, Kot et al., 1994]
Glutaredoxin<br>Glutaredoxin-like NrdH	Reduce dehydroascorbate, peroxiredoxins, and methionine sulfoxide reductase. Reduced non-enzymatically by glutathione.	*grxA*,,<br>*grx*1, *grx*C2,<br>*nrd*H,<br>BBMN68_1397,	*B. longum* BBMN68,	[Elias et al., 2008, Xiao et al., 2011, Kwak et al.,2016]
Peroxideroxin (alkyl hydroperoxide reductase subunit C)	Reduces H_2O_2, organic peroxides and peroxynitrite	ahpC,<br>BL0615	*B. longum* NCC2705	[Xiao et al., 2011, Zuo et al., 2014, Klijn et al., 2005]
Peroxideroxin<br>OsmC family	Peroxidase activity with a strong preference for organic hydroperoxides	BLI010_09070	*B. infantis JCM 7010*	[Dubbs et al., 2007]
Peroxiredoxin Q/BCP	Protein reduces and detoxifies hydroperoxides, shows substrate selectivity toward fatty acid hydroperoxides.	LTBL16_ 000976,<br>BL0615	*B. longum* LTBL16,<br>*B. longum* NCC2705,	[Kwak et al.,2016, Oberg, et al., 2016, Huang et al., 2020]
Thioredoxin	Reduction of disulfide bonds of other proteins by cysteine thiol-disulfide exchange	*trxA,B,*<br>BBMN68_991,<br>BLD 0988	*B. longum* BBMN68,<br>*B. longum* NCC2705	[Kwak et al.,2016, Lu et al.,2014, Xiao et al., 2011, Serata et al., 2012, Jia et al.,2017]
Thioredoxin reductese	Reduction of oxidized thioredoxins and glutaredoxin -like NrdH protein .	*trx*B,<br>BL0164	*B. longum* BBMN68,<br>*B. longum* LTBL16,<br>*B. longum* NCC2705,<br>*B. animalis lactis*	[Oberg, et al., 2016, Lu et al., 2014, Xiao et al., 2011, Klijn et al., 2005, Huang et al., 2020, Jia et al.,2017]
NAD(P)H oxidase	Source of cellular reactive oxygen species, transfer electrons from NADPH to oxygen molecule.	*nox,*<br>LTBL16_ 001911	*B. longum* LTBL16,	[O'Callaghan et al.,2016, Lu et al., 2014, Xiao et al., 2011, Serata et al., 2012, Jia et al.,2017]
Dihydroorotate oxidase	Generates H_2O_2-forming NADH	*pyrK,*	*B. bifidum* CNCMI-4319	[Kawasaki et al.,2009, Jia

	oxidase activity and indirect production of H_2O_2.	CNCMI_0917, *pyrD* CNCMI_0378		et al.,2017]
Oxygen-dependent coproporphyrinogen III oxidase	Involved in detoxifying molecular oxygen and/or H_2O_2		*B. animalis lactis*	[Ruiz et al., 2012]
Possible Class I pyridine nucleotide-disulfideoxidoreductase (PNDR)	Involve in the cellular oxidative stress response	BL1626,	*B. longum* NCC 2705, *B. longum* BBMN68,	[Xiao et al., 2011, Delcardayre et al., 1998]
P-type ATPase	Transport of manganese to the bacterial cell	*mntP*, zntA1 BBMN68_1149	*B. longum* BBMN68	[Xiao et al., 2011, Jia et al.,2017]
Ferritin; ferroxidase; DNA starvation/stationary phase protection protein	Enzymes catalyzes the oxidation of Fe2+ ions by hydrogen peroxide, which prevents hydroxyl radical production by the Fenton reaction	*dps,*	*B. longum* NCC2705	[Klijn et al., 2005, Zhai,et al., 2019]
DsbA family oxidoreductase	Catalyzes intrachain disulfide bond formation as peptides emerge into the cell's periplasm	*dsbA,*	*B. longum MCC00353*	[Zhai,et al., 2019] BioCyc
Ribonucleotide reductase	DNA oxidative damage-protective protein	*nrd*A, BL1752	*B. longum* BBMN68, *B. longum* D2957	[Xiao et al., 2011, Oberg, et al., 2016]
Nucleotide triphosphate pyrophosphohydrolases	DNA oxidative damage-protective proteins	*mut*T1	*B. longum* BBMN68	[Xiao et al., 2011]
NAD-dependent protein deacetylase of SIR2 family	Involved in the response to oxidative stress. NAD + -dependent deacetylation of σH and transcription factor FOXO3a. Improve foxo-dependent transcription of antioxidant enzymes and reduce ROS levels in cells	*sir*2, LTBL16_ 002010	*B. longum* LTBL16	[Halsey et al., 2004, Guo et al., 2017]

Linoleic acid isomerase	Partcipates in linoleic acid metabolism. Conjugated linoleic acid metabolites exhibit the ability to protect cells from oxidative effects.	*lai*, CNCMI4319_0491	*B. bifidum CNCM I-4319*	[Macdonald et al., 2000]
Cyclopropane-fatty-acyl-phospholipid synthase	Catalyzes cyclopropane fatty acid (cell-surface component) biosynthesis	BBMN68_1705,	*B. longum* BBMN68,	[Xiao et al., 2011]
Hydroxycinnamic acid esterase	Release of hydroxycinnamates from plant-based dietary sources	*cae*A	*B. longum*	[Kelly et al., 2018]
S-adenosylhomocysteinase, S-ribosylhomocysteinase	Synthesizes cysteine from methionine using homocysteine as an intermediate	*ahc*Y, *lux*S, FC12_GL001705	*Bifidobacterium* spp.,	[Wada et al., 2021]
Subtilisin-like serine protease, cell envelope protease	Catalyzes the cleavage of peptide bonds	*aprE*, *cep*	*B. longum* KACC91563	[Chang et al., 2013]

References

Jia, F.-F.; Zhang, L.-J.; Pang, X.-H.; Gu, X.-X.; Abdelazez, A.; Liang, Y.; Sun, S.-R.; Meng, X.-C. Complete genome sequence of bacteriocin-producing Lactobacillus plantarum KLDS1.0391, a probiotic strain with gastrointestinal tract resistance and adhesion to the intestinal epithelial cells. *Genome*. **2017,** 109, 432–437.

Kwak, W.; Kim, K.; Lee, C.; Lee, C.; Kang, J.; Cho, K.; Yoon, S.H.; Kang, D.K.; Kim, H.; Heo, J.; et al. Comparative analysis of the complete genome of Lactobacillus plantarum GB-LP2 and potential candidate genes for host immune system en-hancement. *J. Microbiol. Biotechnol*. **2016**, 26, 684–692.

Kot, E.; Haloftis, G.; Bezkorovainy, A. Iron accumulation by bifidobacteria at low pO2 and in air: Action of putative ferrox-idase. *J. Agric. Food Chem*. **1994**, 42, 685–688.

O'Callaghan, A.; van Sinderen, D. Bifidobacteria and their role as members of the human gut microbiota. Front. Microbiol. 2016, 7, 925

Elias, R.J.; Kellerby, S.S.; Decker, E.A. Antioxidant activity of proteins and peptides. *Crit. Rev. Food Scien. Nutr*. **2008,** 48, 430–441.

Xiao, M.; Xu, P.; Zhao, J.; Wang, Z.; Zuo, F.; Zhang, J.; Ren, F.; Li, P.; Chen, S.; Ma, H. Oxidative stress-related responses of Bifidobacterium longum subsp. longum BBMN68 at the proteomic level after exposure to oxygen. *Microbiology*. **2011**, 157, 1573–1588.

Zuo, F.; Yu, R.; Khaskheli, G.B.; Ma, H.; Chen, L.; Zeng, Z.; Mao, A.; Chen, S. Homologous overexpression of alkyl hydroperoxide reductase subunit C (ahpC) protects Bifidobacterium longum strain NCC2705 from oxidative stress. *Res. Microbiol***. 2014**, 165, 581–589.

Klijn, A.; Mercenier, A; Arigoni, F. Lessons from the genomes of Bifidobacterial. *FEMS Microbiol. Rev.* **2005**, 29, 491–509.

Dubbs, J.M.; Mongkolsuk, S. Peroxiredoxins in Bacterial Antioxidant Defense. *Subcell. Biochem*. **2007**, 44, 143–193.

Oberg, T.S.; Broadbent, J.R. Hydrogen Peroxide Resistance in Bifidobacterium Animalis Subsp. Lactis and Bifidobacterium Longum. In Stress and Environmental Regulation of Gene Expression and Adaptation in Bacteria, II; *de Bruijn, F.J., Ed.; Wiley: New York, NY, USA*, **2016**; 2, 638–656.

Huang, G.; Pan, H.; Zhu, Z.; Li, Q. The complete genome sequence of Bifidobacterium longum LTBL16, a potential probiotic strain from healthy centenarians with strong antioxidant activity. *Genome.* **2020**, 112, 769–773.

Serata, M.; Iino, T.; Yasuda, E.; Sako, T. Roles of thioredoxin and thioredoxin reductase in the resistance to oxidative stress in Lactobacillus casei. *Microbiology.* **2012**, 158, 953–962.

Kawasaki, S.; Satoh, T.; Todoroki, M.; Nimura, Y. b-Type Dihydroorotate Dehydrogenase Is Purified as a H_2O_2-Forming NADH Oxidase from Bifidobacterium bifidum. *Appl. Environ. Microbiol.* **2009**, 75, 629-636.

Ruiz, L.; Gueimonde, M.; Ruas-Madiedo, P.; Ribbera, A.; de los Reyes-Gavilán, C.G.; Ventura, M.; Margolles, A.; Sánchez, B. Molecular clues to understand the aerotolerance phenotype of Bifidobacterium animalis subsp. lactis. *Appl. Environ. Microbiol*. **2012**, 78, 644–650.

Delcardayre, S.B.; Davies, J.E. Staphylococcus aureus coenzyme A disulfide reductase, a new subfamily of pyridine nucleotide-disulfide oxidoreductase. Sequence, expression, and analysis of cdr. *J. Biol. Chem*. **1998**, 273, 5752–5757.

Oberg, T.S.; Warda, R.E.; Steele, J.L.; Broadbent, J.R. Transcriptome analysis of Bifidobacterium longum strains that show a differential response to hydrogen peroxide stress. *J. Biotechn*. **2015**, 212, 58–64.

Halsey, T.A.; Vazquez-Torres, A.; Gravdahl, D.J.; Fang, F.C.; Libby, S.J. The Ferritin-Like Dps Protein Is Required for Salmonella enterica Serovar Typhimurium Oxidative Stress Resistance and Virulence. Amer. Soc. Microbiol. Infect. Immun. 2004, 72, 1155–1158.

Guo, Q.; Li, S.; Xie, Y.; Zhang, Q.; Liu, M.; Xu, Z.; Sun, H.; Yang, Y. The NAD+-dependent deacetylase, Bifidobacterium longum Sir2 in response to oxidative stress by deacetylating SigH (σH) and FOXO3a in Bifidobacterium longum and HEK293T cell respectively. *Free Radic. Biol. Med*. **2017**, 108, 929–939.

MacDonald H.B. Conjugated Linoleic Acid and Disease Prevention: A Review of Current Knowledge. *J. Amer. Coll. of Nutr*. **2000**, 19, 1115-1185.

Kelly, S.M.; O'Callaghan, J.; Kinsella, M.; Van Sinderen, D. Characterisation of a Hydroxycinnamic Acid Esterase from the Bifidobacterium longum subsp. longum Taxon. *Front. Microbiol*. **2018**, 9, 2690.

Wada, M.; Fukiya, S.; Suzuki, A.; Matsumoto, N.; Matsuo, M.; Yokota, A. Methionine utilization by bifidobacteria: Possible existence of a reverse transsulfuration pathway. *Biosci. Microbiota Food Health.* **2021**, 40, 80–83.

Chang, O.; Seol, K.-H.; Jeong, S.-G.; Oh, M.-H.; Park, B.-Y.; Perrin, C.; Ham, J.-S. Casein hydrolysis by Bifidobacterium longum KACC91563 and antioxidant activities of peptides derived therefrom. *J. Dairy Sci*. **2013**, 96, 5544–5555.

Table S2. Catalog the orthologs antioxidant genes of *B. longum*

Enzyme name	Number of orthologs
Alkyl hydroperoxide reductase AhpC	6
Alkyl hydroperoxide reductase AhpF	6
Class I pyridine nucleotide-disulfide oxidoreductase	3
Cobalamin-independent methionine synthase II	9
DSBA oxidoreductase	9
Dihydroorotate dehydrogenase	9
Glutaredoxin grxC2 (nrdH)	7
Glutathione import ATP-binding protein GsiA	8
Hydroxycinnamic acid esterase CaeA - carboxylesterase A	4
Linoleic acid isomerase	5
NADH oxidase	11
Oxygen-dependent coproporhpyrinogen III oxidase	9
P-type ATPase	9
Peptidase O pepO	9
Permease	5
Peroxiredoxin	5
Polyphosphate kinase Ppk	9
Pyrophosphohydrolase MutT1	9
Ribonucleotide reductase NrdA	6
Subtilisin-like serine protease	2
Superoxide dismutase SodB	12
Superoxide dismutase SodC	11
Thioredoxin	4
Thioredoxin domain protein	9
Thioredoxin peroxidase	7
Thioredoxin reductase	14
Thioredoxin reductase-like protein	6

Table S3. Distribution genes of antioxidant functionality in the genomes of *B. longum*

**Strain**	**Alkyl hydroperoxide reductase AhpC**	**Alkyl hydroperoxide reductase AhpF**	**Class I pyridine nucleotide disulfideoxido reductase**	**DSBA oxidoreductase**	**Dihydroorotate dehydrogenase**	**Glutaredoxin grxC2 (nrdH)**	**Glutathione import ATP-binding protein GsiA**	**Linoleic acid isomerase**	**NADH oxidase**	**P-type ATPase**
B. longum DJO10A	+	+	+	+	+	+	+	+	+	+
B. longum isolate MGYG-HGUT-01292	+	+	+	+	+	+	+	+	+	+
B. longum NCC2705	+	+	+	+	+	+	+	+	+	+
B. longum strain 105-A	+	+	+	+	+	+	+	+	+	+
B. longum strain 35624	+	+	+	+	+	+	+	+	+	+
B. longum strain 51A	+	+	+	+	+	+	+	+	+	+
B. longum strain BAMA-B05	+	+	+	+	+	+	+	+	+	+
B. longum strain BG7	+	+	+	+	+	+	+	+	+	+
B. longum strain BIM B-813D	+	+	+	+	+	+	+	+	+	+
B. longum strain BXY01	+	+	+	+	+	+	+	+	+	+
B. longum strain CACC 517	+	+	-	+	+	+	+	+	+	+
B. longum strain HN001	+	+	+	+	+	+	+	+	+	+
B. longum strain Jih1	+	+	+	+	+	+	-	+	+	+
B. longum strain JSRL02	+	+	+	+	+	+	+	+	+	+
B. longum strain K15	+	+	-	+	+	+	+	+	+	+
B. longum strain K5	+	+	-	+	+	+	+	+	+	+
B. longum strain LC67	+	+	+	+	+	+	+	+	+	+
B. longum strain LTBL16	+	+	+	+	+	+	+	+	+	+
B. longum strain NCTC11818	+	+	+	+	+	+	+	+	+	+
B. longum strain ZJ1	+	+	+	+	+	+	+	+	+	+
B. longum subsp. *longum* BBMN68	+	+	+	+	+	+	+	+	+	+
***B. longum* subsp. *longum* GT15**	**+**	**+**	**+**	**+**	**+**	**+**	**+**	**+**	**+**	**+**
B. longum subsp. *longum* JCM 1217	+	+	+	+	+	+	+	+	+	+
B. longum subsp. *longum* JDM301	+	+	+	+	+	+	+	+	+	+
B. longum subsp. *longum* KACC 91563	+	+	+	+	+	+	+	+	+	+

B. longum subsp. *longum* strain AH1206	+	+	+	+	+	+	+	+	+	+
B. longum subsp. *longum* strain BORI	+	+	+	+	+	+	+	+	+	+
B. longum subsp. *longum* strain CCUG30698	+	+	+	+	+	+	+	-	+	+
B. longum subsp. *longum* strain JCM 11340	+	+	+	+	+	+	+	+	+	+
B. longum subsp. *longum* strain JCM 11341	+	+	+	+	+	+	+	+	+	+
B. longum subsp. *longum* strain JCM 11342	+	+	+	+	+	+	+	+	+	+
B. longum subsp. *longum* strain JCM 11343	+	+	+	+	+	+	+	+	+	+
B. longum subsp. *longum* strain JCM 7050	+	+	+	+	+	+	+	+	+	+
B. longum subsp. *longum* strain JCM 7052	+	+	+	+	+	+	+	+	+	+
B. longum subsp. *longum* strain JCM 7053	+	+	+	+	+	+	+	+	+	+
B. longum subsp. *longum* strain KCTC 3128	+	+	+	+	+	+	+	+	+	+
B. longum subsp. *longum* strain KCTC 3421	+	+	+	+	+	+	+	+	+	+
B. longum subsp. *longum* strain KCTC 5914	+	+	+	+	+	+	+	+	+	+
B. longum subsp. *longum* strain NCIMB8809	+	+	+	+	+	+	+	+	+	+

Table S3 (Continued). Distribution genes of antioxidant functionality in the genomes of *B. longum*

**Strain**	**Permease**	**Peroxi redoxin**	**Subtilisin-like serine protease**	**Thioredoxin domain protein**	**Thioredoxin peroxidase**	**Thioredoxin reductase**	**Thioredoxin reductase-like protein**
B. longum DJO10A	-	+	+	+	+	+	+
B. longum isolate MGYG-HGUT-01292	+	+	-	+	+	+	+
B. longum NCC2705	-	+	+	+	+	+	+
B. longum strain 105-A	-	+	+	+	+	+	+
B. longum strain 35624	-	+	+	+	+	+	+
B. longum strain 51A	-	+	+	+	+	+	+
B. longum strain BAMA-B05	-	+	+	+	+	+	+
B. longum strain BG7	-	+	+	+	+	+	+
B. longum strain BIM B-813D	-	+	+	+	+	+	+
B. longum strain BXY01	-	+	-	+	-	+	+

B. longum strain CACC 517	-	+	+	+	+	+	+
B. longum strain HN001	-	+	+	+	+	+	+
B. longum strain Jih1	-	+	+	+	+	+	+
B. longum strain JSRL02	-	+	+	+	+	+	+
B. longum K15	-	+	+	+	+	+	+
B. longum strain K5	-	+	+	+	+	+	+
B. longum strain LC67	-	+	+	+	+	+	+
B. longum strain LTBL16	-	+	+	+	+	+	+
B. longum strain NCTC11818	-	+	+	+	+	+	+
B. longum strain ZJ1	-	+	+	+	+	+	+
B. longum subsp. longum BBMN68	-	+	+	+	+	+	+
***B. longum* subsp. *longum* GT15**	**-**	**+**	**+**	**+**	**+**	**+**	**+**
B. longum subsp. *longum* JCM 1217	-	+	+	+	+	+	+
B. longum subsp. *longum* JDM301	-	+	-	+	-	+	+
B. longum subsp. *longum* KACC 91563	-	+	+	+	+	+	+
B. longum subsp. *longum* strain AH1206	-	+	+	+	+	+	+
B. longum subsp. *longum* strain BORI	-	+	+	+	+	+	+
B. longum subsp. *longum* strain CCUG30698	-	+	+	+	+	+	+
B. longum subsp. *longum* strain JCM 11340	-	+	+	+	+	+	+
B. longum subsp. *longum* strain JCM 11341	-	+	+	+	+	+	+
B. longum subsp. *longum* strain JCM 11342	-	+	+	+	+	+	+
B. longum subsp. *longum* strain JCM 11343	-	+	+	+	+	+	+
B. longum subsp. *longum* strain JCM 7050	-	+	+	+	+	+	+
B. longum subsp. *longum* strain JCM 7052	-	+	+	+	+	+	+
B. longum subsp. *longum* strain JCM 7053	-	+	+	+	+	+	+
B. longum subsp. *longum* strain KCTC 3128	-	+	+	+	+	+	+
B. longum subsp. *longum* strain KCTC 3421	+	+	+	+	+	+	+
B. longum subsp. *longum* strain KCTC 5914	-	+	+	+	+	+	+
B. longum subsp. *longum* strain NCIMB8809	-	+	+	+	+	+	+
B. longum subsp. *longum* strain YS108R	-	+	+	+	+	+	+

Table S4. Parameters of the analyzed transcriptomic samples

Sample name	Raw samples		Trimmed samples	
	Size, mln reads	Size, blnb.p.	Size, mln reads	Size, blnb.p.
BL1	30.0	3.0	28.7	2.5
BL2	32.2	3.2	30.7	2.7
BL3	26.6	2.7	24.5	2.3
BL4	30.5	3.1	29.1	2.6
BL5	30.4	3.1	29.2	2.6

Table S5. The total number of upregulated transcripts in cells of *B. longum* GT15 under the action of a certain oxidative stress factor

Total transcripts*	Response to H_2O_2	Response to O_2 2 h	Response to O_2 4 h
Response to H_2O_2	**190**	52	52
Response to O_2 2 h	58	**117**	52
Response to O_2 4 h	72	99	**177**

* results are expressed as more 2-fold change after oxidative stress compared to control (untreated) cells

Table S6. The total number of detected proteins in increased level in cells of *B. longum* GT15 under the action of a certain oxidative stress factor

Total proteins*	Response to H_2O_2	Response to O_2 2 h	Response to O_2 4 h
Response to H_2O_2	**13**	5	3
Response to O_2 2 h	5	**19**	13
Response to O_2 4 h	3	13	**14**

* results are expressed as more 2-fold change after oxidative stress compared to control (untreated) cells

Table S7. The total number of common transcripts and proteins in increased level in cells of *B. longum* GT15 under the action of a certain oxidative stress factor

Total proteins/transcripts*	Response to H_2O_2	Response to O_2 2 h	Response to O_2 4 h
Response to H_2O_2	**8**	2	3
Response to O_2 2 h	2	**14**	10
Response to O_2 4 h	3	10	**13**

* results are expressed as more 2-fold change after oxidative stress compared to control (untreated) cells

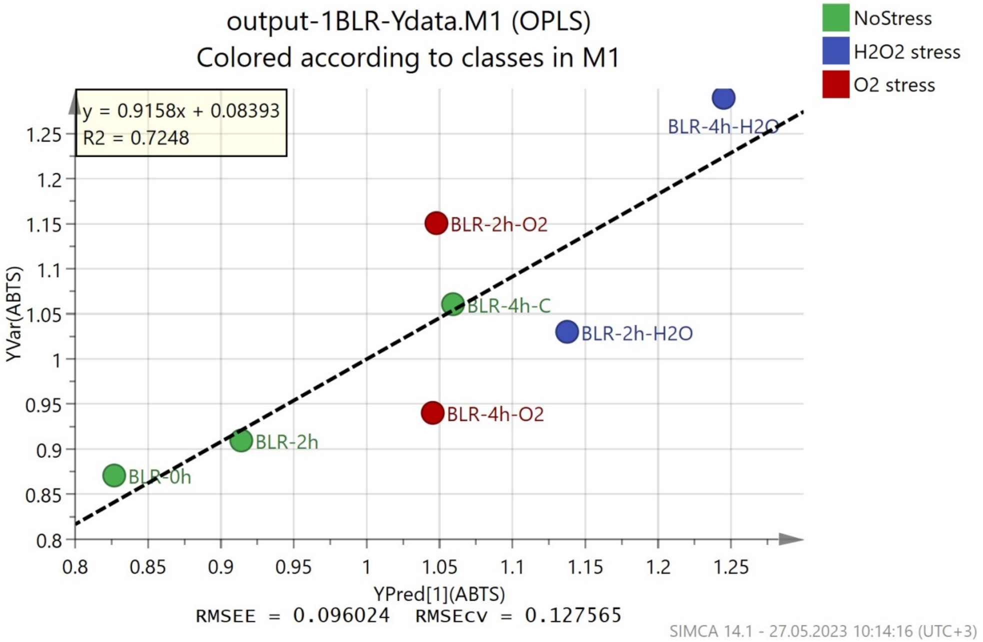

Figure S1. OPLS regression models on metabolic fingerprints of *B. longum* GT15 cells and their antioxidant activity (ABTS•+ method).

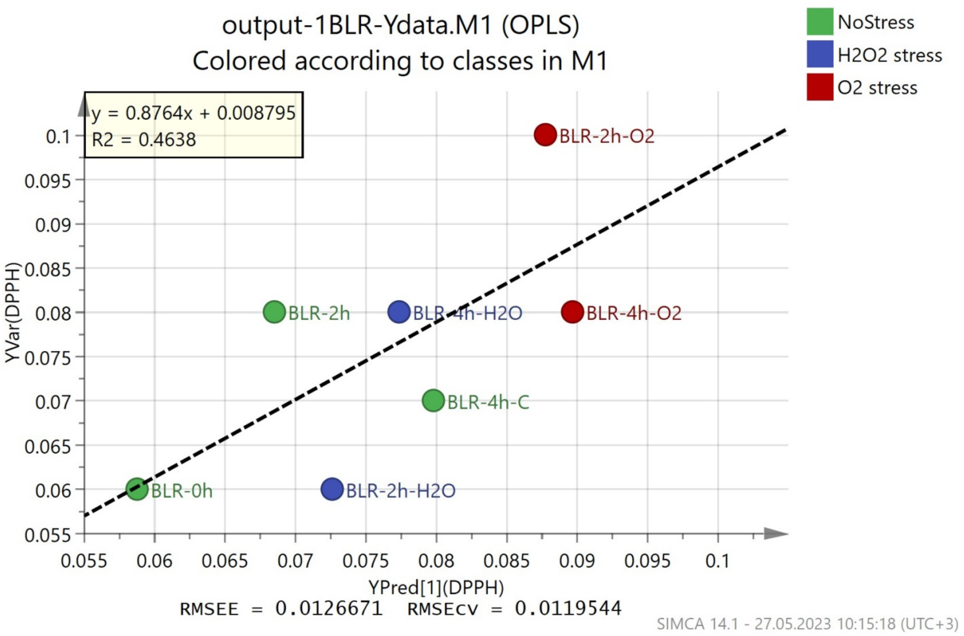

Figure S2. OPLS regression models on metabolic fingerprints of *B. longum* GT15 cells and their antioxidant activity (DPPH• method).

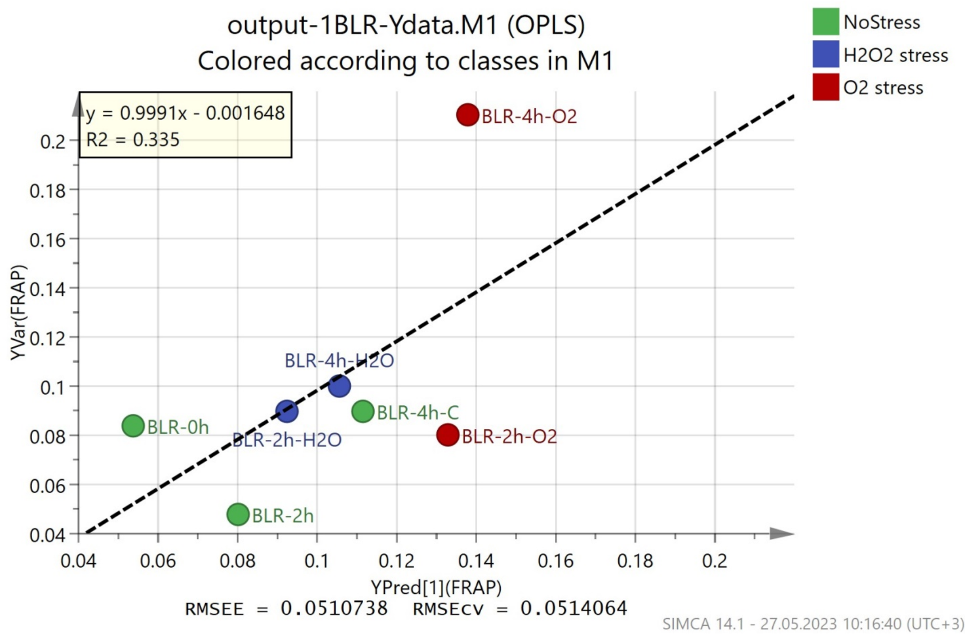

Figure S3. OPLS regression models on metabolic fingerprints of *B. longum* GT15 cells and their antioxidant activity (FRAP method).
